# Fabrication of Ni-silicide/Si heterostructured nanowire arrays by glancing angle deposition and solid state reaction

**DOI:** 10.1186/1556-276X-8-224

**Published:** 2013-05-10

**Authors:** Hsun-Feng Hsu, Wan-Ru Huang, Ting-Hsuan Chen, Hwang-Yuan Wu, Chun-An Chen

**Affiliations:** 1 Department of Materials Science and Engineering, National Chung Hsing University, Taichung 40227, Taiwan

**Keywords:** Silicide/Si heterostructured nanowire arrays, Glancing angle deposition, Size-dependent phase formation

## Abstract

This work develops a method for growing Ni-silicide/Si heterostructured nanowire arrays by glancing angle Ni deposition and solid state reaction on ordered Si nanowire arrays. Samples of ordered Si nanowire arrays were fabricated by nanosphere lithography and metal-induced catalytic etching. Glancing angle Ni deposition deposited Ni only on the top of Si nanowires. When the annealing temperature was 500°C, a Ni_3_Si_2_ phase was formed at the apex of the nanowires. The phase of silicide at the Ni-silicide/Si interface depended on the diameter of the Si nanowires, such that epitaxial NiSi_2_ with a {111} facet was formed at the Ni-silicide/Si interface in Si nanowires with large diameter, and NiSi was formed in Si nanowires with small diameter. A mechanism that is based on flux divergence and a nucleation-limited reaction is proposed to explain this phenomenon of size-dependent phase formation.

## Background

Free-standing heterostructure nanowire arrays have been widely investigated for their applications in nano gas sensors [1], nano photocatalysts [2-4], and field emission devices [5]. Covering the semiconductor nanowire arrays with metal particles can improve their sensitivity as gas sensors because metal particles on the surfaces of nanowires induce the formation of Schottky barrier junctions. The adsorption and desorption by the analysts alter the overall resistance of the nanowire [1]. For photocatalyts, the Schottky junction at the semiconductor/metal interface can enhance the separation of photoexcited electron–hole pairs to improve the efficiency of photocatalytic degradation of organic pollutant [2-4].

Metal silicides have been widely applied in Si technology as ohmic contacts, low-resistivity interconnects, and Schottky barrier, and they have been introduced into Si nanowires. The most common method for forming silicide/Si nano-heterojunctions is to drive thermally silicidation of Ni [6-12], Co [13], Pt [14], and Mn [15]. These silicide/Si heterostructured nanowires have been used in nanoscale devices [16]. Large-area silicide/Si heterostructured nanowire arrays have the potential to be used in field emission devices [5], gas sensors, or photocatalysts. However, such studies are very rare in previous publications.

The phase formation between the metal and Si is critical to microelectronics as well as nanoelectronics. Silicide selection is related to many factors, such as temperature of formation, the orientation and size of the Si nanowires, and the process of Ni proving [9-11]. This study presents a distinctive method for fabricating large-area Ni-silicide/Si heterostructured nanowire arrays by combining nanosphere lithography, metal-induced catalytic etching, glancing angle deposition, and solid state reaction. A size-dependent phase formation at the silicide/Si interface was observed, and a mechanism was provided.

## Methods

**Figure 1:**
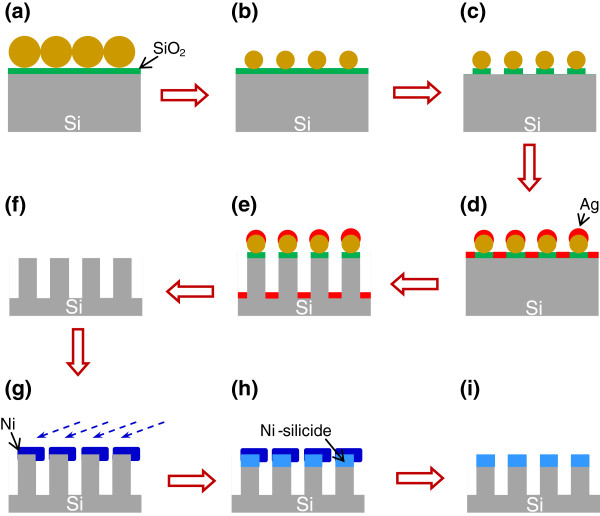
**Schematic illustration of the procedure for the fabrication of Ni-silicide/Si heterostructured nanowire arrays on Si(100) substrates.** (**a**) Spread close packed monolayer PS spheres array on SiO2/Si(100) substrate, (**b**) O2 plasma etching, (**c**) Ar plasma etching, (**d**) Ag deposition, (**e**) metal-induced catalytic etching, (**f**) Ag, PS spheres and SiO2 removing, (**g**) glancing angle Ni deposition, (**h**) rapid thermal annealing treatment, and (**i**) Ni removing.

N-type Si(100) substrates with a resistivity of 1 to 10 Ω cm were cut into 1 × 2 cm^2^ pieces. Figure 1 shows a schematic illustration of the procedure for the fabrication of Ni-silicide/Si heterostructured nanowire arrays on Si(100) substrates. The substrates were cleaned using the standard RCA (Radio Corporation of America) procedure and then immersed into boiling solutions of H_2_SO_4_:H_2_O_2_ = 3:1 for 10 min to form a hydrophilic oxide layer. A close-packed monolayer array of polystyrene (PS) spheres with mean diameter of 202 nm was formed on the substrate by the drop-casting method [17]. The diameter of PS spheres was reduced by O_2_ plasma, and then, the exposed oxide layer was removed by Ar plasma. A 20-nm gold thin film was deposited on the patterned substrate. The samples were etched by immersing in the mixture solutions of HF, H_2_O_2_ and deionized water (HF = 5 M and H_2_O_2_ = 0.176 M) at 50°C for 3 min. An ordered silicon nanowire arrays were achieved after removing the residual PS spheres and gold film by the tetrahydrofuran (THF) and HNO_3_ solution, respectively. Before being loaded into the deposition chamber, the sample was dipped in a dilute HF solution to remove the oxide layer on the surface. The evaporation beam has a 20° incident angle with respect to the substrate surface. After 100-nm Ni film being deposited on top of Si nanowire arrays, the samples were annealed by rapid thermal annealing at 500°C for 4 min in a forming gas (N_2_:H_2_ ratio, 95:5). The unreacted Ni coats were removed by immersing the samples in the HNO_3_ solution. The morphologies of samples were observed using field emission scanning microscopy (FESEM, JEOL JSM-6700F, Akishima-shi, Japan) with an accelerated voltage of 3 kV. The crystal structures of nanowires in a JEOL JEM-2100F operating at 200 kV were verified using transmission electron microscopy (TEM) analysis.

## Results and discussion

**Figure 2:**
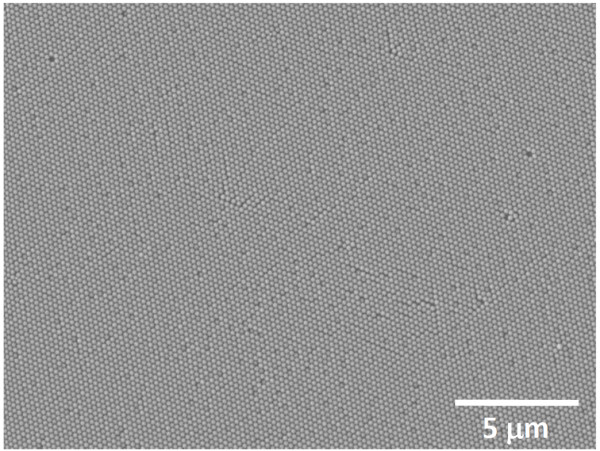
**Low-magnification SEM image of a close-packed monolayer array of PS sphere on SiO**_**2**_**/Si(100) substrate formed by drop-casting method.**

Figure 2 shows the low-magnification SEM image of a close-packed monolayer array of PS spheres on Si substrate, formed by the drop-casting method. The variation in the size of the PS spheres caused the monolayer of PS spheres to have a few stacking faults and point defects.

**Figure 3:**
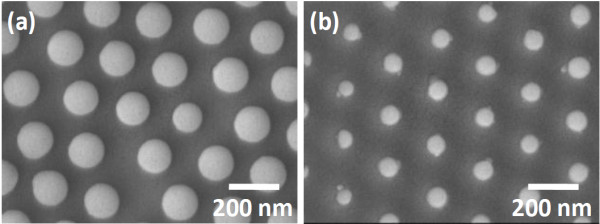
**FESEM images of close-packed monolayer PS sphere arrays.** With various diameter fabrication by (**a**) 3-min (sample A) and (**b**) 6-min O_2_ (sample B) plasma etching and then Ar plasma etching.

The diameter of Si nanowires that were fabricated by combining PS sphere lithography with Ag-induced catalytic etching was controlled by varying the size of PS spheres [18]. Figure 3 shows the FESEM image of a closed-packed monolayer of PS spheres with various sizes that were fabricated by O_2_ plasma etching for different periods. The PS spheres with diameters of 150 ± 8 and 81 ± 8 nm were prepared by O_2_ etching for 3 and 6 min, respectively. Sample A referred to the former, and sample B referred to the latter.

**Figure 4:**
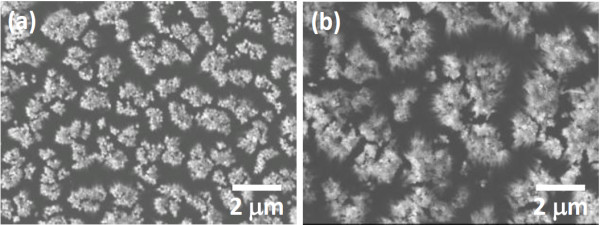
**Top view FESEM images of Si nanowires.** Formed by immersing the 20-nm Ag coated (**a**) sample A and (**b**) sample B in HF/H_2_O_2_ solution at 50°C for 3 min.

**Figure 5:**
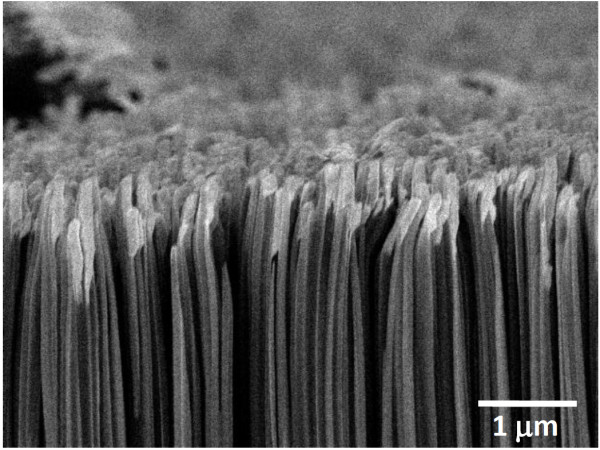
Cross section FESEM images of a Si nanowire array after glancing angle Ni deposition.

Following Ag-induced catalytic etching for 3 min, the Si nanowires were 5- to 6-μm long. Surface tension and van der Waals forces were responsible for the bunching of the tops of the Si nanowires, as shown in Figure 4. Figure 5 shows the SEM image of the cross section of a Si nanowire array after glancing angle Ni deposition, which indicated that Ni was only deposited on top of Si nanowires.

**Figure 6:**
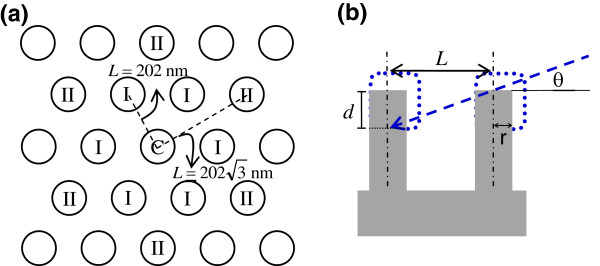
**Illustrations of the Si nanowires arrays.** (**a**) Top view illustration and (**b**) cross section illustration.

In an ideal situation, the Si nanowires are well aligned without bunching. The depth of Ni deposition is discussed as follows. Figure 6a shows an illustration of the top view of Si nanowire array. Each nanowire, marked C, is surrounded by six nearest nanowires, marked I, and six second nearest ones, marked II. These neighboring Si nanowires act as shadowing centers and cause the Ni to be deposited only on the top of the nanowires during the glancing angle deposition. For example, when the diameter of nanowires is larger than 100 nm, the I and II Si nanowires completely sheltered the bottom of the C nanowire from the deposition of Ni. Figure 6b shows an illustration of the cross-sectional Si nanowires, and the length of the Ni-coated part of the Si nanowire can be estimated as:

d=Ltanθ

where *d* is the length of the Ni-coated part, *L* is the distance between two Si nanowires, and *θ* is the incident angle of Ni deposition. The length of the Ni-coated part is about 74 nm when shadowed by I nanowires and about 127 nm when shadowed by II nanowires. In fact, length fluctuations were observed, as shown in Figure 5, because the bunching of the Si nanowires changed the distance between them.

**Figure 7:**
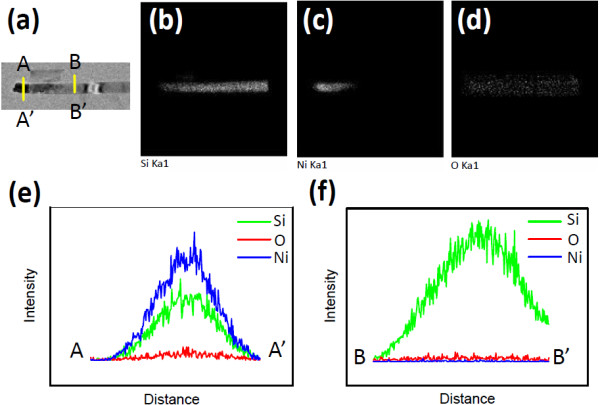
**TEM image of an example of Ni-silicide/Si heterostructured nanowire and corresponding EDS mapping images.** (**a**) TEM image of an example of Ni-silicide/Si heterostructured nanowire and corresponding EDS mapping images of (**b**) Si, (**c**) Ni, and (**d**) O. EDS line profiles along the (**e**) AA' and (**f**) BB' lines indicated in (**a**).

Thermal annealing of the samples at 500°C yielded Ni-silicide/Si heterostructured nanowire arrays. Figure 7 shows an example of a Ni-silicide/Si heterostructured nanowire. EDS mapping data in Figure 7b,c indicate that the Ni signal was only observed at the apex of the nanowire, where the Ni-silicide formed.

**Figure 8:**
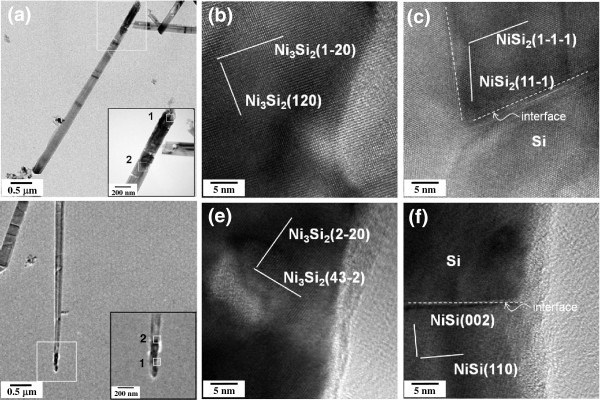
**Phases of Ni-silicide were identified by the analysis of atomic-resolution TEM images.** (**a**) TEM image of a Ni-silicide/Si heterostructured nanowire with large diameter formed from sample A. The insert is the magnified image of the silicide part of nanowire, and the area corresponds to the square in (**a**). (**b**) Atomic resolution TEM image of the front of the silicide part, and the area corresponds to the square 1 in the insert of (**a**). (**c**) Atomic resolution TEM image of the interface of silicide and Si, and the area corresponds to the square 2 in the insert of (**a**). (**d**) TEM image of a Ni-silicide/Si heterostructured nanowire with small diameter formed from B-sample. The insert is the magnified image of the silicide part of nanowire, and the area corresponds to the square in (d). (**e**) Atomic resolution TEM image of the front of the silicide part, and the area corresponds to the square 1 in the insert of (d). (**f**) Atomic resolution TEM image of the interface of silicide and Si, and the area corresponds to the square 2 in the insert of (**d**).

The phases of Ni-silicide were identified by the analysis of atomic-resolution TEM images, as shown in Figure 8. Based on the results of the analysis results, two forms of Ni-silicide were identified. The Si nanowires with large diameter were formed from sample A, in which the phase at front of Ni-silicide part was Ni_3_Si_2_ and that at the Ni-silicide/Si interface was NiSi_2_. NiSi_2_ grew epitaxially in the Si nanowires and had a {111} facet at the interface. However, Si nanowires with small diameter were formed from sample B, in which the phase at front of the Ni-silicide part was also Ni_3_Si_2_ and that at the Ni-silicide/Si interface was NiSi.

**Figure 9:**
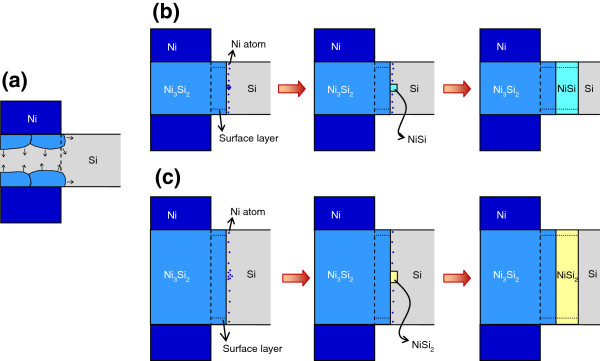
**Schematic illustrations of the mechanism of two-stage silicidation at the apex of Si nanowire.** (**a**) A schematic illustration of the initial stage of silicidation. (**b**) A schematic illustration of the second stage silicidation in the Si nanowire with small diameter. (**c**) A schematic illustration of the second stage silicidation in the Si nanowire with large diameter.

The mechanism of silicide formation at the apex of Si nanowire is two-stage silicidation. In the initial stage, as shown in Figure 9a, silicide grows in the radial direction, which is similar to the solid state reaction of metal film with a Si layer. The phase selection between metal and Si couples depends strongly on the atomic ratio of Ni/Si. This dependence is observed not only in the thin film reactions [19] but also in the nanoparticle reactions [20]. In this study, the apex of Si nanowires covered with a considerable number of Ni atoms, which can be regarded as a system with a high Ni/Si atomic ratio, causing the formation of a metal-rich phase (Ni_3_Si_2_) at the Ni-coated part of Ni-silicide.

In the second stage, the Ni silicide axially intruded into the Si nanowire from the Ni-coated part located at the front of the nanowire. Such penetration of Ni silicide involves different thermally activated processes, such as the volume, surface, and interface diffusions of Ni. In this study, the phase selection depended on the diameter of the Si nanowires, such that NiSi_2_ and NiSi were observed in nanowires with large diameters and small diameters, respectively. The reasons for this phenomenon are discussed as follows.

First, the location of silicide nucleation in the Si nanowires in the axial direction is discussed. Wu et al. [11] studied the formation of Ni silicide in the Si nanowires through point and line contact reaction. By the point contact reaction between Ni nanodots and a Si nanowire, the nucleation and growth of NiSi grains start at the middle of the point contacts. By the line contact reaction between PS nanosphere-mediated Ni nanopatterns and a Si nanowire, silicide growth starts in the contact area. Wu et al. concluded that the mechanism of silicide growth in Si nanowires is based on the basis of flux divergence. Lu et al. [21] obtained the similar results for the formation of Pt silicide in the Si nanowires. They also performed molecular dynamic simulations to support the experimental results: a low atom flux of Pt caused the dissolution and distribution of Pt in the Si nanowire. Then, the nucleation of a silicide can occur between the two contacts where the Pt atoms dissolve, and the most probable site of nucleation is the middle because of the buildup of concentration that occurs in the middle. Second, the position of nucleation of silicide in Si nanowires in the radial direction is discussed. Chou et al. studied the growth of NiSi [22] and NiSi_2_[23] in Si nanowires by *in situ* high-resolution TEM. They found that the silicidation reaction is nucleation-limited, and both NiSi and NiSi_2_ atomic layers grew from the center rather than from the edge in the epitaxial interfaces in Si nanowires. Conclude the nucleation of silicide in Si nanowires as shown above. When the flux of metal atom is low, the metal dissolves into Si and become distributed in the Si nanowire or at the silicide/Si interface; the nucleation of silicide then occurs where the concentration of metal reaches the required supersaturation concentration.

Figure 9b,c schematically depict the second stage of silicide formation. After the initial stage of Ni-silicide formation, Ni diffusion occurs chiefly along the silicide surface toward a Si/silicide interphase boundary, because volume diffusion is much slower than the diffusion of Ni along the silicide surface [24], causing Ni atoms to dissolve into Si from the outer silicide interface. Owing to low atom flux, Ni atoms distribute into the Si part at the Si/silicide interface, and the nucleation of silicide can then occur anywhere at the Si/silicide interface but most probably occurs in the middle [21-23].

The processing temperature window of NiSi for the formation of silicide thin film by solid state reaction is from 300°C to 750°C [25]. In this study, the annealing temperature is 500°C, so the formation of NiSi is expected. However, why does the NiSi_2_ form in the Si nanowire with large diameter? Assume that the atom flux through the outer silicide interface is the same for nanowires with large and small diameters. The concentration of Ni in the middle of Si/silicide interface decreases as the diameter of nanowire increases. In nanowires with large diameter, the concentration of Ni does not reach the supersaturation required for the nucleation of NiSi but it does reach that required for the nucleation of NiSi_2_, NiSi_2_ nucleates. Oppositely, in nanowires with small diameter, NiSi nucleates.

## Conclusions

In this study, ordered Si nanowire array samples were fabricated by nanosphere lithography and metal-induced catalytic etching, and then, Ni-silicide/Si heterostructured nanowire arrays were obtained by glancing angle Ni deposition and solid state reaction. The front of Ni-silicide part of nanowires was metal-rich phase (Ni_3_Si_2_) because the apex of the Si nanowires that was coated by Ni deposition had high Ni/Si atomic ratio. The Ni-silicide at the Ni-silicide/Si interface in Si nanowires with large diameter was epitaxial NiSi_2_ with an {111} facet and that in Si nanowires with small diameter was NiSi. A mechanism that is based on flux divergence and a nucleation-limited reaction is proposed to explain this phenomenon of phase formation that depends on the size of the nanowire.

## Competing interests

The authors declare that they have no competing interests.

## Authors' contributions

HFH supervised the overall study, discussed the results, and wrote the manuscript. WRH fabricated the Ni-silicide/Si heterostructured nanowire arrays and analyzed the results. THC performed TEM measurement. HYW performed SEM measurement. CAC helped in the analysis of TEM results. All authors read and approved the final manuscript.
